# Risk mapping of scrub typhus infections in Qingdao city, China

**DOI:** 10.1371/journal.pntd.0008757

**Published:** 2020-12-02

**Authors:** Hualei Xin, Peng Fu, Junling Sun, Shengjie Lai, Wenbiao Hu, Archie C. A. Clements, Jianping Sun, Jing Cui, Simon I. Hay, Xiaojing Li, Zhongjie Li

**Affiliations:** 1 Division of Infectious Disease, Qingdao City Center for Disease Control and Prevention, Qingdao, Shandong, China; 2 Key Laboratory of Surveillance and Early Warning on Infectious Disease, Division of Infectious Disease, Chinese Center for Disease Control and Prevention, Beijing, China; 3 World Health Organization (WHO) Collaborating Centre for Infectious Disease Epidemiology and Control, School of Public Health, Li Ka Shing Faculty of Medicine, The University of Hong Kong, Hong Kong, China; 4 Department of Anesthesiology, Qingdao Fuwai Cardiovascular Hospital, Qingdao, Shandong, China; 5 School of Geography and Environmental Science, University of Southampton, Southampton 1BJ, United Kingdom; 6 School of Public Health, Fudan University, Key Laboratory of Public Health Safety, Ministry of Education, Shanghai, China; 7 School of Public Health and Social Work, Institute of Health and Biomedical Innovation, Queensland University of Technology, Queensland, Australia; 8 Faculty of Health Sciences, Curtin University, Bentley, Western Australia, Australia; 9 Institute for Health Metrics and Evaluation, University of Washington, Seattle, WA, United States of America; Faculty of Science, Ain Shams University (ASU), EGYPT

## Abstract

**Background:**

The emergence and re-emergence of scrub typhus has been reported in the past decade in many global regions. In this study, we aim to identify potential scrub typhus infection risk zones with high spatial resolution in Qingdao city, in which scrub typhus is endemic, to guide local prevention and control strategies.

**Methodology/Principal findings:**

Scrub typhus cases in Qingdao city during 2006–2018 were retrieved from the Chinese National Infectious Diseases Reporting System. We divided Qingdao city into 1,101 gridded squares and classified them into two categories: areas with and without recorded scrub typhus cases. A boosted regression tree model was used to explore environmental and socioeconomic covariates associated with scrub typhus occurrence and predict the risk of scrub typhus infection across the whole area of Qingdao city. A total of 989 scrub typhus cases were reported in Qingdao from 2006–2018, with most cases located in rural and suburban areas. The predicted risk map generated by the boosted regression tree models indicated that the highest infection risk areas were mainly concentrated in the mid-east and northeast regions of Qingdao, with gross domestic product (20.9%±1.8% standard error) and annual cumulative precipitation (20.3%±1.1%) contributing the most to the variation in the models. By using a threshold environmental suitability value of 0.26, we identified 757 squares (68.7% of the total) with a favourable environment for scrub typhus infection; 66.2% (501/757) of the squares had not yet recorded cases. It is estimated that 6.32 million people (72.5% of the total population) reside in areas with a high risk of scrub typhus infection.

**Conclusions/Significance:**

Many locations in Qingdao city with no recorded scrub typhus cases were identified as being at risk for scrub typhus occurrence. In these at-risk areas, awareness and capacity for case diagnosis and treatment should be enhanced in the local medical service institutes.

## Author summary

Scrub typhus is a bacterial zoonosis caused by Orientia tsutsugamushi, which is mostly carried by rodents and chigger mites, and occasionally transmitted to humans by bites of infected larval mites. Scrub typhus is geographically endemic across broad areas of south and southeastern Asia, the Pacific islands and northern Australia. In recent years, an obvious increasing trend of the incidence of this disease has been noticed all around the world. China is one of the most endemic country for this disease, with an approximate 10-fold increase of the annual case number from 2006 to 2013. In this study, by using a prediction model for infectious diseases, we found that the highest infection risk area was located at the mid-east and northeast regions of Qingdao, with gross domestic product and precipitation might developed the greatest impact for the occurrence of scrub typhus. Furthermore, 68.7% of the region in Qingdao might have a suitable environment for the occurrence of scrub typhus, with most of them hadn’t reported cases yet. This study highlights the emergency to enhance the awareness of this disease and improve the capacity of diagnosis and treatment among medical institutions in high risk areas in Qingdao.

## Introduction

Scrub typhus is a vector-borne disease mainly caused by *Orientia tsutsugamushi* (Ot), which is an obligate, intracellular bacterium [1]. *Orientia tsutsugamushi* is transmitted by the bite of infected mites. The lifecycle of mites consists of an egg, two six-legged stages and four eight-legged stages. Larval mites, often called chiggers, are the only life stage that feeds on humans and transmits *O*. *tsutsugamushi*. Clinical features in human patients can range from mild (asymptomatic) to fatal and are generally flu-like, with symptoms of fever, headache and myalgia. During severe infection, complications such as meningitis, intravascular complications, severe pneumonitis/peritonitis, and/or cardiac distress have been reported [2,3]. The mortality rate varies and can reach 50% [4,5]. An eschar at the site of chigger feeding is a classic clinical feature of scrub typhus; however, the spotted fever group of rickettsioses can also include an inoculation eschar at the bite site, adding an additional challenge during differential diagnosis [1]. The drug of choice for the treatment of scrub typhus is doxycycline (tetracycline, chloramphenicol, and azithromycin have also been used successfully) [6–8]; however, the emergence of antibiotic-resistant strains of *O*. *tsutsugamushi* is of concern [9]. Additionally, there are no long-lasting, broadly protective vaccines available against scrub typhus [10].

Scrub typhus was thought to be confined geographically to the Asia-Pacific areas, bounded by Japan in the east, Pakistan in the west, Russia in the north and Australia in the south [11]. Recently [12], there has been tremendous widespread re-emergence of scrub typhus in locations such as India, Micronesia, and the Maldives, where it had been forgotten, and its incidence is increasing in locations such as South Korea and China north of the Yangtze River, where it was previously unknown [13]. Additionally, sporadic scrub typhus cases have been identified in countries and regions outside the traditional “tsutsugamushi triangle” in the Asia-Pacific region, such as Chile, the United Arab Emirates (UAE) and Africa [14]. A recent study found that mutations or the introduction of new strains may explain the re-emergence of scrub typhus in an epidemic form [15,16]. The World Health Organization has dubbed scrub typhus one of the world’s most underdiagnosed/underreported diseases; it often requires hospitalization, highlighting the necessity for an improved understanding of the disease [17]. In China, scrub typhus remains a serious public health problem, and an increasing trend has been identified in recent years. In 2006, scrub typhus was added to the national infectious disease surveillance system as a voluntarily reportable disease. In 2009, the national guidance for scrub typhus has been published, which required that scrub typhus cases should be reported in the national surveillance system within 24 hours as soon as a scrub typhus case was diagnosed in the medical institutions. Shandong Province is one of the main endemic areas in northern China, and the first scrub typhus case in northern China was found in Shandong Province. During 2014–2015, enhanced surveillance measures for scrub typhus had been implemented in Shandong Provinces, including strengthened case report, case epidemiological investigation and sampling.

Knowledge of the geographical distribution and burden of scrub typhus is essential for determining the optimal allocation of limited resources necessary for scrub typhus control. In recent years, the use of mapping and spatial analyses to better understand disease outbreaks, distribution of associated vectors, and potential predictive factors have been employed for rickettsial and other vector-borne diseases [18–22]. Risk mapping for scrub typhus occurrence in at-risk areas has previously been performed at the national level in China [23], but the distribution of the disease at high spatial resolutions, particularly in northern China, remains poorly characterized. In this study, we predict the risk of scrub typhus considering a 3 km × 3 km spatial resolution in Qingdao city, Shandong Province, China, using a boosted regression tree (BRT) model based on reported scrub typhus data and a range of environmental and socioeconomic covariates, with the aim of providing precise insights into the public health burden imposed by scrub typhus.

## Methods

### Study area

Qingdao city located at the middle-east of China, covers approximately 11,000 km^2^ area, with 3 urban counties and 7 sub-urban counties. The population reached approximately 8.70 million people in 2018, with a population density of 790 people/km^2^.The mean annual average temperature is ~12.7°C, the mean annual average precipitation is ~662 mm, the mean annual average sunshine hours is ~2,125 hours, the gross domestic product (GDP) is ~1,100 billion Chinese Yuan (CNY) (156 billion US dollar) and the per capita GDP is ~0.1 million Chinese Yuan (CNY) (15,473 US dollar) (Statistics Bureau of Qingdao, 2018).

### Data collection and ethical considerations

In this study, we collated all clinically diagnosed and laboratory-confirmed cases of scrub typhus in Qingdao from 1 January 2006 to 31 December 2018 from the Chinese Center for Disease Control and Prevention (China CDC). Case information (including demographic information, illness onset time, diagnosis and report time, and reporting institution) was reported by clinicians to the web-based National Notifiable Infectious Disease Reporting Information System (NNIDRIS) of the China CDC. Nine hundred eighty-nine cases were collected and validated for the final analysis.

The National Health and Family Planning Commission of China determined that the collection of human scrub typhus case data is considered continuing public health surveillance of infectious diseases and is exempt from institutional review board assessment. All data were supplied and analysed in an anonymous format, without access to personal identifying information.

### Data analyses

**Fig 1:**
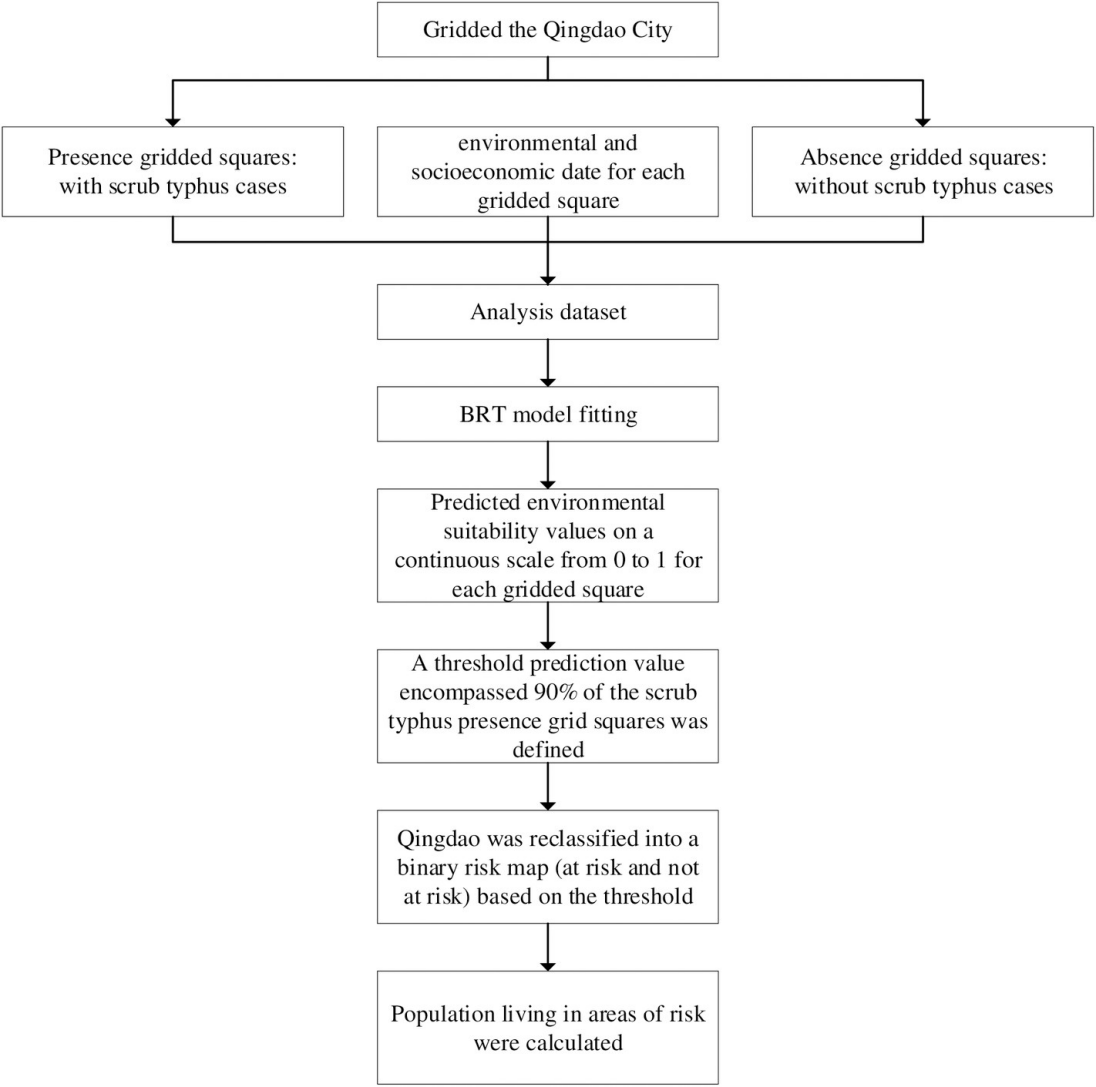
Flow chart of the model analysis.

To map the environmental suitability for scrub typhus infection in humans, we applied a BRT approach to establish a multivariate empirical relationship between the probability of occurrence of the disease and the environmental conditions in locations where the disease has been reported [24–26]; this required the generation of 1) a comprehensive compendium of known locations of disease occurrence in humans (presence data); 2) a set of background points representing locations where scrub typhus has not yet been reported (absence data); and 3) a set of high-resolution globally gridded environmental and socioeconomic covariates hypothesized to affect scrub typhus infection risk. We estimated the relative risk of infection for each 3 × 3 grid square (1,101 grid squares in total) across Qingdao (Fig 1).

A total of 379 spatial coordinates were assigned to 989 scrub typhus cases. In brief, presence was defined as a 3 × 3 grid square with at least one spatial coordinate located within it; in these squares, the risk label was set to 1, while a grid square with no spatial coordinate was considered absence and labelled 0. In total, the final dataset contained 257 unique presence and 514 randomly selected absence grid squares, resulting in a 1:2 ratio for presence and absence grid squares in the dataset.

A set of eight 1 km × 1 km gridded data surface layers of environmental and socioeconomic variables hypothesized to influence the distribution of scrub typhus infection in humans was assembled [13,14,23,27,28]. These variables included the following: 1) annual average temperature; 2) annual cumulative sunshine hours; 3) gross domestic product (GDP); 4) annual cumulative precipitation; 5) elevation; 6) urban accessibility; 7) normalized difference vegetation index (NDVI); and 8) urbanization.

The meteorological variables (temperature and sunshine hours) were collected from the China Meteorological Data Service Center (CMDC) (http://data.cma.cn). Elevation, GDP and the NDVI were extracted from the Resource and Environment Data Cloud Platform website (www.resdc.cn/Default.aspx). The approximately 1 km × 1 km gridded urban accessibility dataset, which estimates the travel time to a city of 50,000 or more people, was obtained from the European Commission Joint Research Center (http://forobs.jrc.ec.europa.eu/). Urbanization was characterized by urban and non-urban areas; urban areas were defined as those with a population density ≥1,000 people per km^2^, and non-urban areas were defined as those with <1,000 people per km^2^ [29]. The 1 km × 1 km gridded population dataset from the 2010 census in China was used to calculate the population density for each grid square (www.resdc.cn/Default.aspx).

The covariate values were extracted from the raster maps above and were averaged across the grids containing every 3 km × 3 km grid square.

The random selection of absence data was performed 300 times, with each iteration involving the construction of a dataset including the 257 “presence” grid squares and 514 randomly selected “absence” grid squares [23]. After each random selection process, we constructed 771 samples, dividing the samples into two parts. In this study, training samples and test samples accounted for 75% (578) and 25% (193) of the total samples (771). Then, we fitted a BRT model for each dataset. Each of the 300 models predicted environmental suitability on a continuous scale from 0 to 1, with a final prediction map generated by calculating the mean prediction across all models for each 3 km × 3 km grid square. Each of the 300 BRT models was fitted using gbm. step subroutine in the dismo package in the R statistical programming environment (version 3.4.1, R Foundation for Statistical Computing, Vienna, Austria). The main tuning parameters were as follows: tree. complexity = 5, learning. rate = 0.001, bag. fraction = 0.5, cv. folds = 10, max. trees = 10,000. The other tuning parameters of the algorithm were maintained at their default values [25]. A ten-fold cross-validation method was applied to each model to prevent over-fitting, and area under the curve (AUC) statistics were used to evaluate the predictive performances of the BRT models in terms of how well the model predicted the presence/absence of the disease in each grid cell.

Populations living in areas of risk were estimated by using a threshold probability to reclassify the probabilistic risk maps into a binary risk map (at risk and not at risk); then, the total and county-level populations in the “at-risk” areas considering a gridded population dataset from 2010 was extracted. The threshold value encompassed 90% of the scrub typhus presence grid squares [26,30]. The study area was subsequently partitioned into locations that had suitable environments but had not yet reported scrub typhus cases, had suitable environments and had reported scrub typhus cases, and unsuitable environments. ArcGIS software (version 10.2.2, ESRI, Redlands, CA, USA) was used to plot the data and model outputs.

## Results

### Spatiotemporal patterns

A total of 989 scrub typhus cases were recorded in the 9 counties in Qingdao city. The median age of the cases was 58 (IQR: 49~66) years, and the ratio of males to females was 0.8:1. Nine hundred and fifty-seven cases (96.8%) were found in October and November. There was an average of only 44 scrub typhus cases (annual average incidence rate: 0.47/100 000) reported each year during 2006–2013, but this value increased to 128 cases per year during 2014–2018 (annual average incidence rate: 1.36/100 000). In 2014 and 2015, when active local enhanced surveillance was carried out, the number of reported cases was 197 per year, which is approximately 4-fold higher than the number of reported cases per year (54 cases for average) during the period with no active surveillance programme.

**Fig 2:**
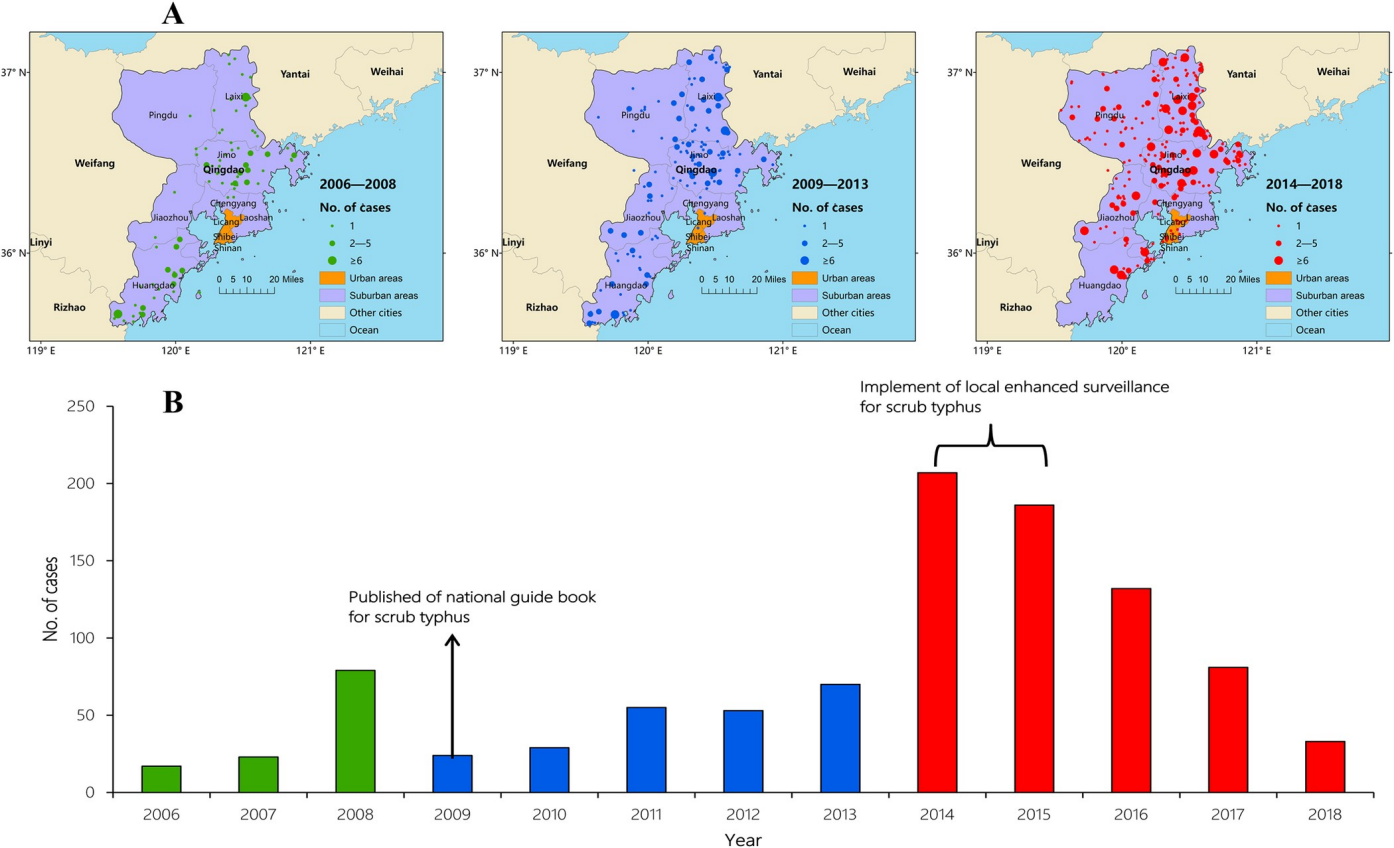
A) Map showing the distribution of the 379 spatial coordinates of scrub typhus records in Qingdao city. The locations are classified by year of occurrence to show those that occurred (i) between 2006–2008, before the publication of the national guide book for scrub typhus; (ii) between 2009–2013, after the publication of the national guide book for scrub typhus; and (iii) between 2014–2018, after the implementation of enhanced surveillance for scrub typhus in Qingdao city. B) The number of scrub typhus cases reported in Qingdao city over time [31].

Fig 2A shows the 379 locations with scrub typhus cases in the Qingdao area, stratified by year (2006–2008, 2009–2013 and since 2014). This map is accompanied by Fig 2B, which shows the number of reported cases by year. These figures highlight that most case records (97.2%, 962/989) and spatial coordinates (93.7%, 355/379) were located in the suburban areas of Qingdao, and the spatial correlation was strong (Moran’s *I* = 0.131, p<0.01).

### Modelled distribution of scrub typhus

**Fig 3:**
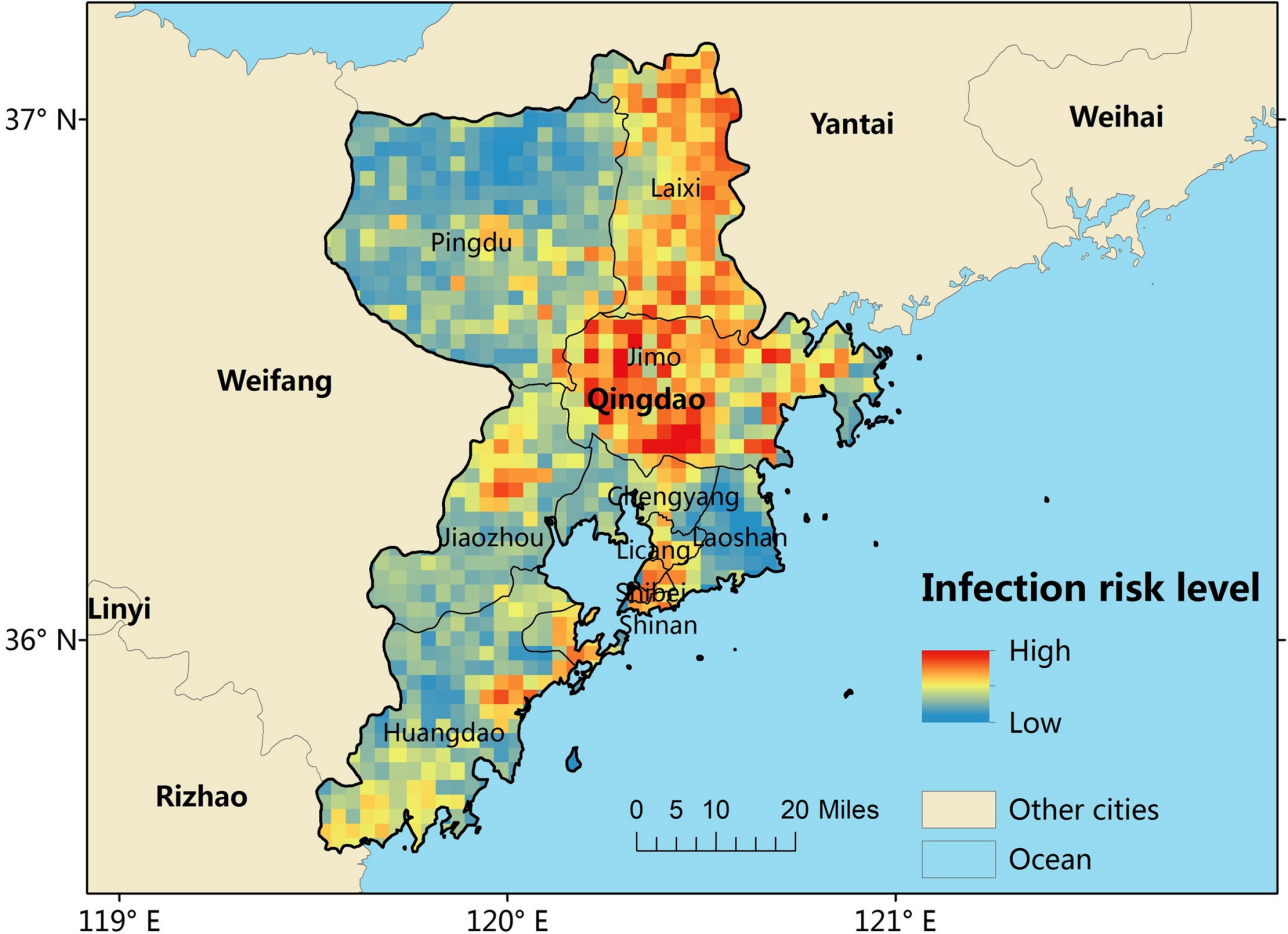
Maps of environmental suitability for scrub typhus in Qingdao city [31].

Fig 3 shows the mean of 300 ensemble BRT models. The predicted map reveals that the predicted highest scrub typhus infection risk areas were mainly concentrated in the mid-east and northeast areas of Qingdao. Additionally, small parts of the south and west areas were predicted to be at increased risk for scrub typhus.

**Fig 4:**
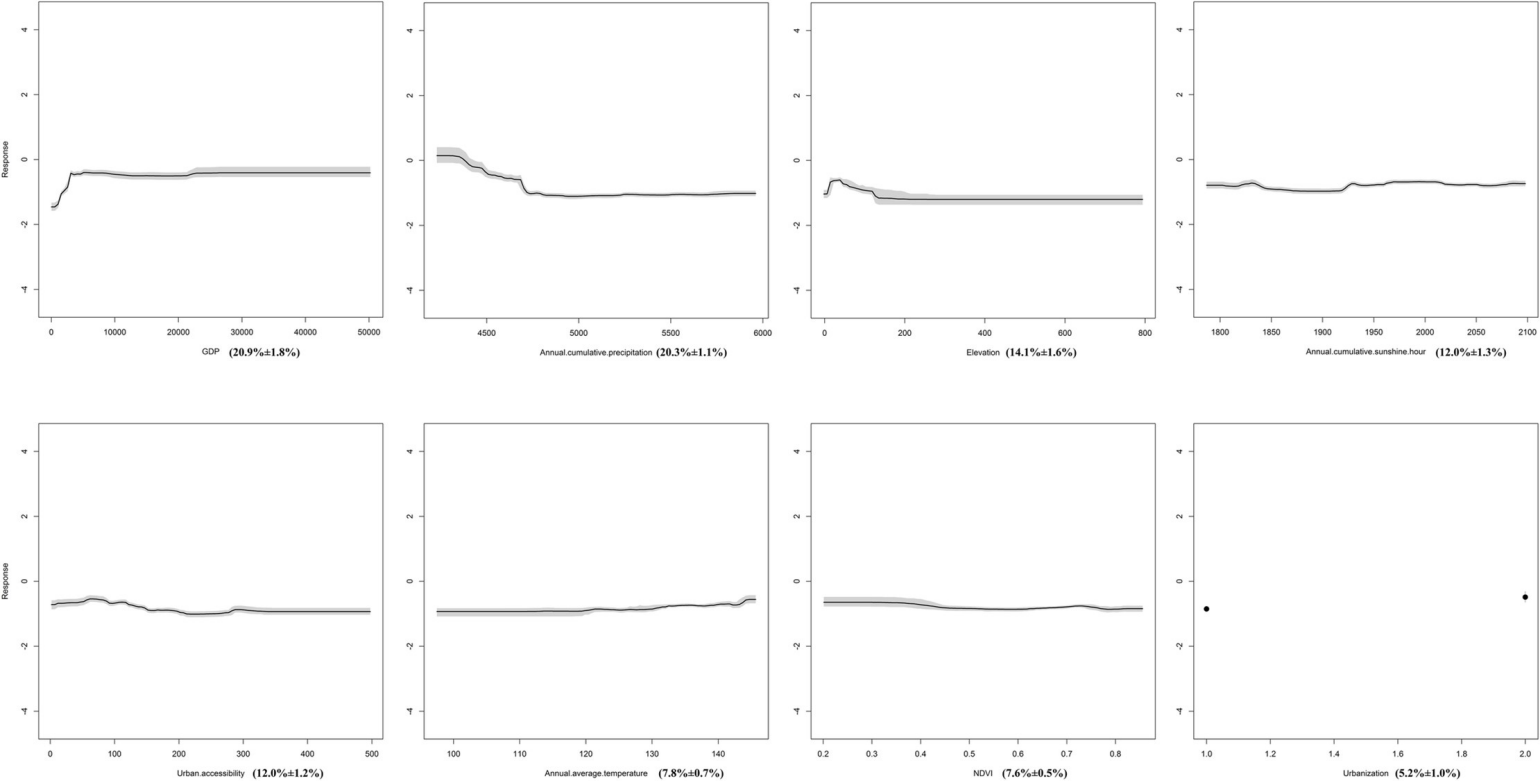
Marginal Effect Curves of Each Predictor.

Table 1 showed the basic characteristics of the environmental and socioeconomic covariates in the 1,101 grid squares. Around the eight covariates, GDP, annual cumulative precipitation, urban accessibility and urbanization experienced statistical difference (*p<0*.*01*) between grid squares with and without scrub typhus spatial coordinate. Fig 4 showed that GDP and annual cumulative precipitation was the most important predictor variable in the model, accounting for 20.9%±1.8% standard error (s.e.) and 20.3%±1.1% s.e. of the variation explained by the ensemble BRT models with a strong association with scrub typhus presence. These were followed by elevation (14.1%±1.6% s.e.), annual cumulative sunshine hours (12.0%±1.3% s.e.), urban accessibility (12.0%±1.2% s.e.), annual average temperature (7.8%±0.7% s.e.), NDVI (7.6%±0.5% s.e.) and urbanization (5.2%±1.0% s.e.) (Fig 4). Validation statistics indicated that the BRT models had high predictive performance, with AUC of 0.852 ± 0.018 s.e. and 0.84 ± 0.012 s.e. for validation data and training data respectively.

**Table 1:** Characteristics of environmental and socioeconomic covariates for overall, with and without scrub typhus spatial coordinate.

Characteristics	Overall (N = 1101)	With scrub typhus spatial coordinate (N = 256)	Without scrub typhus spatial coordinate (N = 845)	*P* value **
GDP (Chinese Yuan, median, IQR*)	2482 (1526–4250)	3155 (2159–5247)	2256 (1361–3957)	<0.01
**Annual cumulative precipitation (mm, median, IQR)**	4804 (4631–5045)	4685 (4568–4917)	4827 (4653–5064)	<0.01
**Elevation (meters, median, IQR)**	56 (16–73)	35 (19–58)	40 (15–77)	0.167
**Annual cumulative sunshine hours (hours, median, IQR)**	1975 (1878–2047)	1979 (1898–2041)	1974 (1877–2048)	0.882
**Urban accessibility (days, median, IQR)**	142 (94–204)	117 (77–173)	150 (102–215)	<0.01
**Annual average temperature (0.1°C, median, IQR)**	133 (129–137)	133 (130–137)	133 (129–137)	0.743
**Normalized difference vegetation index (NDVI)**	0.69 (0.61–0.75)	0.69 (0.59–0.74)	0.69 (0.62–0.76)	0.131
**Urbanization (%)**				
Urban	121 (11)	53 (21)	68 (8)	<0.01
Non-Urban	980 (89)	203 (79)	777 (92)	

*All quantitative covariates in this table were conformed to skew distribution and were described as median (IQR: Inter-Quartile Range).

**Mann Whitney test method was used to compare quantitative covariates between grid squares with and without scrub typhus spatial coordinate; chi-square test was used to compare qualitative covariates between grid squares with and without scrub typhus spatial coordinate.

**Fig 5:**
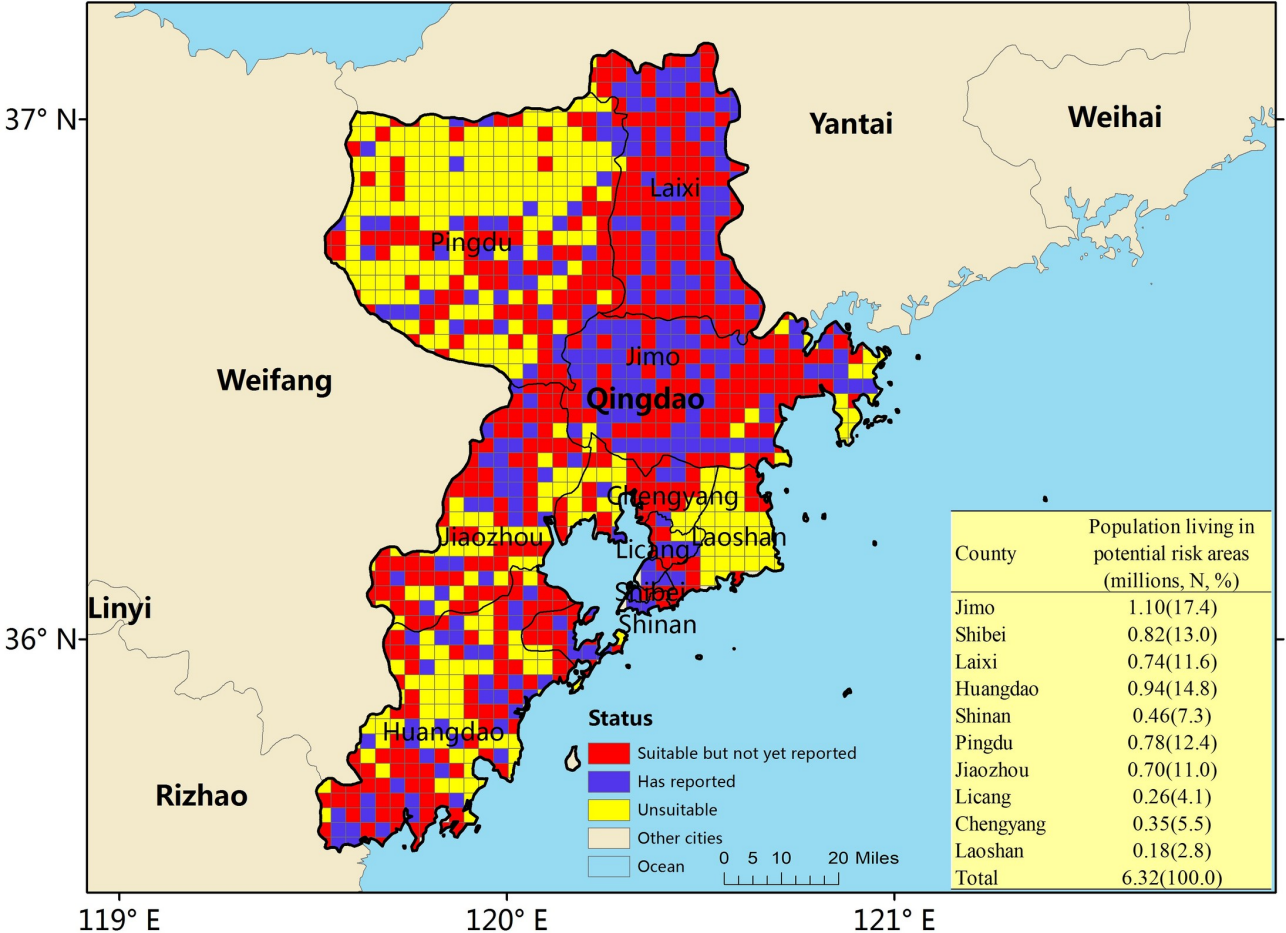
Status of Scrub Typhus Transmission Risk for Each Pixel in Qingdao City. The Red Areas Represent Pixels that are Environmentally Suitable (having a Suitability Value of More than 0.26) but Which have not yet Reported Scrub Typhus Cases in Humans [31].

A threshold environmental suitability value of 0.26 was used to classify each 3 × 3 km grid square on our final map as suitable or unsuitable for scrub typhus infection in humans. A vast majority of Qingdao had a suitable environment for the occurrence of scrub typhus, except for a few areas located in the northwest, southeast and south of Qingdao. Among the 757 grid squares suitable for the occurrence of scrub typhus, 501 (66.2%) had a suitable environment but had not yet reported scrub typhus cases (Fig 5). Furthermore, we summed the populations living in the scrub typhus suitable areas and found that 6.32 million people lived within areas that are environmentally suitable for scrub typhus, accounting for 72.5% of the total population of Qingdao (Fig 5).

## Discussion

In this study, by utilizing a longitudinal surveillance dataset spanning 13 years, we found that most regions of the study area, particularly the mid-east and northeast regions, had a suitable environment for the occurrence of scrub typhus, with GDP and annual average precipitation contributing the most in our model. Additionally, most of the population in Qingdao live in areas that are environmentally suitable for scrub typhus infection.

The predicted risk maps provide a starting point for informing public health activities and indicate areas that have not yet reported cases for control and assessment of disease burden. However, we should recognize that environmental suitability for disease infection in an area does not necessarily mean that it will arrive and/or establish in that location. Rather, our model predicts the potentiality of scrub typhus infection based on the environmental and socioeconomic characteristics of each location. Although local animal reservoir types and density and vector species were not included in our model, the maps provide information on areas where reservoirs and vectors of scrub typhus could colonize.

Previous studies showed that temperature, relative humidity, precipitation, wind speed, and duration of sunshine and cloud cover were positively associated with scrub typhus incidence in several settings, which may be due to the influence of these factors on the existence of chiggers and rodent hosts [14,32]. Additionally, a close association between scrub typhus and vegetation often exists [33]. Endemic foci of scrub typhus are usually in areas of secondary vegetation wherein scrub and grasses provide suitable habitats for vector and rodent hosts [34] and serve as a platform for the parasitic larval stage to attach to passing ground-dwelling vertebrate hosts [35]. Densely vegetated areas close to domestic dwellings are thought to be the main sites of scrub typhus infection exposure [36]. Furthermore, the socioeconomic status variable was chosen to act as a proxy for a variety of important global risk factors for disease, including malnutrition, sanitation quality, and living with domesticated animals [37–41]. A study in South Korea found that changes accompanying urbanization provided suitable habitats (i.e., grasslands and riverbanks) for vectors and small rodents, resulting in urban areas as possible locations for scrub typhus exposure [42]. Our analysis revealed that GDP and annual average precipitation were the most important factors for the occurrence of scrub typhus, which is different from previous studies in southern China, where maximum temperature was found to be the most important factor [23]. This difference might be because meteorological and vegetation factors do not vary much within one city; thus, socioeconomic factors become the main driving force at this scale.

Overall, we predict that over 6 million people live in areas that are environmentally suitable for scrub typhus infection, accounting for 72.5% of the total population in Qingdao. Similar to other vector-borne infectious diseases, scrub typhus may be temporally and spatially variable and, even in the most receptive environments, it is unlikely that all of the population will be infected. The estimates are intended to provide an indicator of the total number of individuals who may require protection during a disease outbreak [26]and aid in prioritizing active surveillance and control. Moreover, these populations should be the focus of efforts to increase awareness and provide guidelines for mitigating personal risk of infection.

The complexity and diversity of scrub typhus transmission cycles involving not only humans but also a multitude of vectors and reservoirs necessitated a modelling approach that accounted for the highly non-linear effects of covariates on the probability of disease presence. The BRT modelling approach is able to do this and has previously been shown to produce highly accurate predictions considering a wide range of species. This ecological niche modelling approach is able to address not only the variation in parasites causing infection but also the various life histories and habitat preferences associated with different vector species [29]. However, it is not possible to use this type of analysis to identify causal links between the covariates and suitability for disease infection [43]; thus, the covariate effects should be carefully interpreted as associations.

Limitations existed in our studies. Our study was recorded from 2006–2018, and many city characteristics, such as the socioeconomic variables might change for such a long period. However, the overall socioeconomic status for different areas in Qingdao city hadn’t changed during our study period, therefore, variables used in our study could reflect the general distribution in Qingdao city. Besides, all the presence data used were collected from a passive disease surveillance system, and the data quality may be influenced by the completeness and accuracy of the data over the studied time period. After implementing enhanced surveillance for scrub typhus in 2014 and 2015, the reported cases increased approximately 4-fold compared to those in other study periods, which might attribute to the strengthened supervise and inspection in the medical institutions and related financial support in the enhanced surveillance project. Therefore, under-reporting is likely a major issue. With a passive reporting system, the under-reporting of surveillance data is inevitable. Therefore, in our study, the BRT model was used to generate risk maps based on data of known cases of scrub typhus and the environment at the locations of the known cases. This information was used to infer the likelihood of scrub typhus presence in other locations without scrub typhus cases according to the surveillance system and can guide the direction of future control measures. Additionally, no data of vectors and rodents were used in our model, which were considered as important factors that could influence the occurrence of scrub typhus, even there might be variables in our model that can act as a proxy.

In this study, we produced the first city-level, high-resolution map of environmental suitability for scrub typhus infection in humans using known reports of disease occurrence and a variety of covariates in a species distribution modelling framework. The predicted risk maps revealed that a large part of Qingdao may be suitable for scrub typhus occurrence; however, in most of these areas, it has not yet been reported. Additionally, a high proportion of the population in Qingdao lives in these areas. Strengthened disease surveillance and public health awareness campaigns should be focused in areas that are highly suitable for scrub typhus occurrence, particularly in areas that are highly suitable but have not yet reported cases.
